# Achieving Sustainable Developments Goal 3 on health from global pharmacy workforce

**DOI:** 10.7189/jogh.10.020350

**Published:** 2020-12

**Authors:** Kayoko Takeda Mamiya, Christopher John, Saja A Alnahar, Lina Bader, Ian Bates

**Affiliations:** 1 Faculty of Pharmaceutical Sciences, Hokkaido University of Science, Sapporo, Hokkaido, Japan; 2 International Pharmaceutical Federation, The Hague, the Netherlands; 3 Faculty of Pharmacy, Yarmouk University, Irbid, Jordan; 4 School of Pharmacy, University College London, London, UK

The United Nation’s (UN) Sustainable Development Goals (SDGs) are designed to ensure that the development of health, education and employment (amongst others) are not only sustainable but also equitable and accessible for everyone [1]. The goal is, by 2030, to stimulate and guide the creation of at least 40 million new jobs in the health and social sectors and to reduce the projected shortage of 18 million health workers, primarily in low- and lower-middle-income countries [1]. A health workforce of an adequate size and skill mix are critical to the attainment of any population health goal. This includes the achievement of universal health coverage, primary health care and the health-related targets of the UN SDGs. However, in the past decade, the issue of health care workers shortage, including pharmacists, has not been resolved in some low-income countries [1-3].

In its response to the global increase in health workers migration and its potential implications, the World Health Organization (WHO) has published its 2010 Global Code of Practice on the International Recruitment of Health Personnel. The code aims to set an international ethical standard in recruiting health workers [2]. In 2016, the WHO established the International Platform on Health Worker Mobility with the aim of advancing cooperation, dialogue and knowledge in the area of health workforce immigration. Moreover, in 2017, the WHO published the National Health Workforce Accounts (NHWA), which aims to standardize health and workforce information systems around the world; the NHWA includes indicators on health workers migration and emigration.

The requirements to achieve good health and well-being (SDG3) and the increasing demand for health workforce require that the capacity of health workers, including pharmacists, meets health needs. Based on that and knowing that pharmacists, mainly community and hospital pharmacists, play a major role in delivering health care services, the role of the pharmacist is undergoing dynamic changes. Therefore, it is essential to understand the current trends in the global pharmacy workforce and the implications of these trends for the future supply of pharmacists since these changes will affect the health workforce in terms of maintaining an adequate size and skill mix. Moreover, surveys of internationally trained pharmacists worldwide have hardly been conducted since 2006, while surveys of internationally trained nurses and doctors are being conducted by the WHO. This paper aims to explain why an international survey on the global internationally trained pharmacy workforce is required now.

## PUSH-PULL FACTORS

To understand the issues related to the migration of the health workforce around the world, we need to know, “Why do health workers migrate to another country?” and “How do they, health workers, decide on the country where they will migrate?”

Professionals’ migration flows respond to a wide range of factors that affect all forms of migration (such as opportunity differentials between sending and receiving countries and historical, political and trade relationships) [4,5]. These factors can be classified as push-factors; reasons why people might want to emigrate, and pull-factors; reasons why a country might seek to attract immigrants (**Table 1**).

**Table 1:** ‘Push’ and ‘pull’ factors influencing health workforce migration [4-6]

‘Push’ factors	‘Pull’ factors
• Low pay (absolute and/or relative)	• Higher pay, opportunities for remittance
• Poor/dangerous working conditions	• Better working conditions
• Unemployment	• Better resourced health system
• Lack of resources	• Career opportunities
• Limited career opportunities	• Provision for post-basic education
• Limited educational opportunities	• Higher standard of living
• Economic/political instability	• Travel opportunities
	• Aid work, political stability

It is clear, however, that combinations of these factors or components of push-pull factors may change over time. In any one migration flow, several complex drivers may interconnect to shape the eventual direction and nature of the movement. In 2018, Nicholas et al. established when and why some drivers are more important than others and are more susceptible to change through external intervention. They modified existing explanations of migration to generate a framework that they called *push-pull plus* [6]. They suggested that migration policy should be understood not simply as a stand-alone lever but within the wider political economy [6].

## ECONOMY AND HEALTH

As indicated for push-pull factors, migration is closely linked to a healthy population, which in turn affects the economic growth and development. In 2013, 20 years after publishing the World Development Report, Lancet Commission, by Jamison et al, revisited the case for investment in health and developed a new investment framework to achieve substantial health gains by 2035 [7]. In a study focussing on lower-middle income countries, Jamison et al report that overall economic growth is associated with a reduction in population mortality [7]. However, the health workforce is unevenly distributed across countries and regions. It is especially scarce in low-income countries where health workers are most needed. In actually, of 57 countries with a critical shortage of health workers, 36 are sub-Saharan African countries [1-3,8,9]. The similar results were shown in the 2012-2013 pharmacist workforce survey reported in 2016 [10].

## STUDENTS’ “INTENTION TO MIGRATE”

**Figure:**
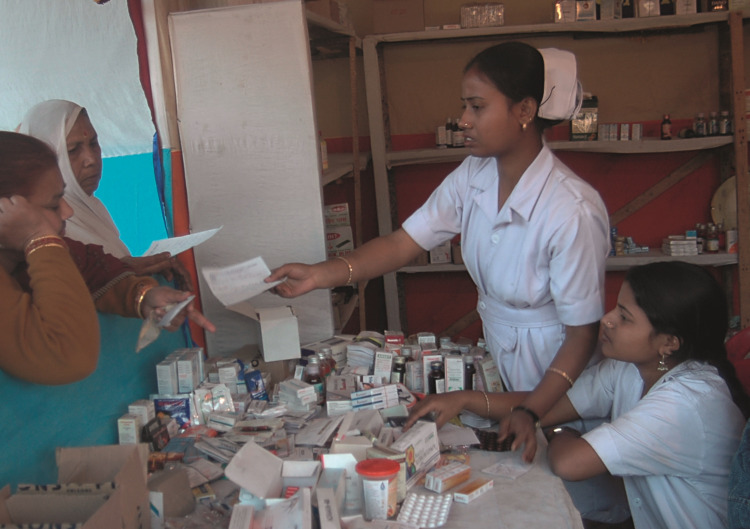
Photo: Pharmacists and pharmacy workers – as medicines experts - are often the first and most accessible point of contact in the health care system.

To improve the unevenly distributed health workforce, it is important to predict future migration trends and survey students’ intentions to migrate. Surveys of students’ intentions to migrate have been undertaken in some countries [4,5,11]. In Ghana, a low-income country, a survey indicated that students saw their pharmacy degrees as just a first step, with further education being a distinct component of their short- or medium-term goals. In addition, the desire to work in a situation in which they and their profession were valued and respected was a prominent point that students expressed in all interviews. Furthermore, most students expressed a commitment to their country, and all expressed a desire to stay in or return to Ghana and usually to contribute to health care. The Ghanaian study indicates that a lack of attention by policy makers and professional bodies to ways of exploiting the contribution of pharmacists to public health may represent a potential human resource loss for health in developing countries [12]. In 2009, an international survey, which investigated the migration intentions of pharmacy students and migration factors that influenced their decisions, showed that in nine countries there was a significant difference between students with no intention to migrate and those with an intention to migrate. In that study, a negative attitude towards the professional and socio-political environment in the home country and a positive perception of opportunities abroad were associated with the intention to migrate, particularly on a long-term basis [11]. On the other hand, the attitudes of students planning short-term migration were not significantly different from those of students who did not intend to migrate. These attitudes, together with gender, knowledge of other migrant pharmacists and past experiences abroad, were associated with an increased propensity for migration [11].

## CONCLUSION

**Figure 1:**
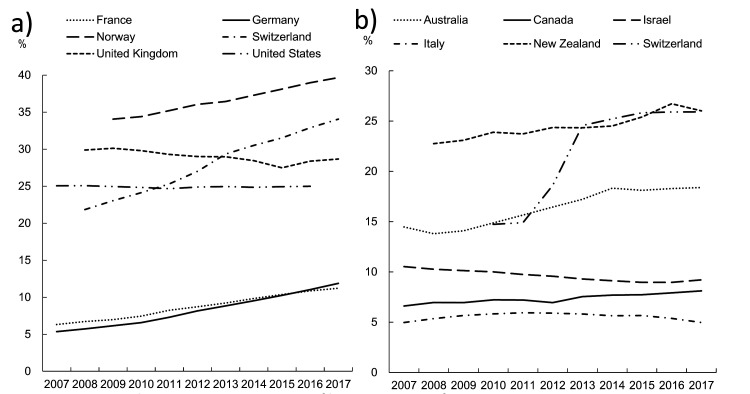
Share of internationally trained (foreign-trained) doctors and nurses. Panel A. Evolution in the share of foreign-trained (internationally trained)doctors, selected OECD countries, 2000 to 2017. Panel B. Evolution in the share of foreign-trained (internationally trained) nurses, selected OECD countries, 2000 to 2017. Source: reference [13].

We receive news about migration almost every day. However, we cannot fully understand or meet the challenges of migration due to the complexity of the associated factors. Migration of the highly skilled health workforce, including pharmacists, is likely to increase in the next ten years (**Figure 1**), although Pharmacists are not included in **Figure 1**. Therefore, there is an urgent need to investigate and understand the migration issues relevant to the pharmacy profession again now.

Access to high-quality health services is vital for improving nations’ health outcomes and for the development of the nations’ economy. Ensuring the availability of an appropriately and sufficiently skilled pharmacy workforce, which are effectively distributed across national health care systems and facilities, is an important approach for improving equitable access to health services, particularly in low- and low-middle-income countries. Despite the fact that pharmacists are playing an important role in access to high-quality health services, there are no sufficient data on internationally trained pharmacists, and limited information has been made available by international organizations. And we will require “quality equivalence” between professionals working in a country – whether they are internationally-trained pharmacists or pharmacists who qualified in their own country. These qualities include all competencies required by pharmacists to provide health care services. Factors affecting migration and the choice of the country of immigration change over time. An uneven distribution of pharmacists and a lack of “quality equivalence” among pharmacists around the world, are likely to be related to the economy, educational system and national policies, as described in the sections “Push-pull factor” and “Students’ intention to migrate”. These issues cannot only be addressed in isolation in individual countries where issues are experienced. Therefore, in order to solve the issues of health workforce migration, which is considered to be the cause of the uneven distribution of health workforce, we need to disseminate and discuss such issues with a broad audience (people who engaged in education, regulation, and practice) and make sense of the available information. Developing a globally shared understanding of factors and issues linked to health workforce migration in general and pharmacy in particular could be achieved through an international survey. The outcomes of this survey should be deployed to improve the distribution of the pharmacist workforce and skill mix worldwide. This report aims to start that process.
